# Increased DNA and RNA damage by oxidation in patients with bipolar I disorder

**DOI:** 10.1038/tp.2016.141

**Published:** 2016-08-09

**Authors:** A S Jacoby, M Vinberg, H E Poulsen, L V Kessing, K Munkholm

**Affiliations:** 1 Psychiatric Center Copenhagen, Rigshospitalet, Faculty of Health and Medical Sciences, University of Copenhagen, Copenhagen, Denmark; 2 Laboratory of Clinical Pharmacology Q7642, Rigshospitalet and Department of Clinical Pharmacology, Bispebjerg Hospital, Faculty of Health and Medical Sciences, University of Copenhagen, Copenhagen, Denmark

## Abstract

The mechanisms underlying bipolar disorder (BD) and the associated medical burden are unclear. Damage generated by oxidation of nucleosides may be implicated in BD pathophysiology; however, evidence from *in vivo* studies is limited and the extent of state-related alterations is unclear. This prospective study investigated for we believe the first time the damage generated by oxidation of DNA and RNA strictly in patients with type I BD in a manic or mixed state and subsequent episodes and remission compared with healthy control subjects. Urinary excretion of 8-oxo-deoxyguanosine (8-oxodG) and 8-oxo-guanosine (8-oxoGuo), valid markers of whole-body DNA and RNA damage by oxidation, respectively, was measured in 54 patients with BD I and in 35 healthy control subjects using a modified ultraperformance liquid chromatography and mass spectrometry assay. Repeated measurements were evaluated in various affective phases during a 6- to 12-month period and compared with repeated measurements in healthy control subjects. Independent of lifestyle and demographic variables, a 34% (*P<*0.0001) increase in RNA damage by oxidation across all affective states, including euthymia, was found in patients with BD I compared with healthy control subjects. Increases in DNA and RNA oxidation of 18% (*P<*0.0001) and 8% (*P*=0.02), respectively, were found in manic/hypomanic states compared with euthymia, and levels of 8-oxodG decreased 15% (*P<*0.0001) from a manic or mixed episode to remission. The results indicate a role for DNA and RNA damage by oxidation in BD pathophysiology and a potential for urinary 8-oxodG and 8-oxoGuo to function as biological markers of diagnosis, state and treatment response in BD.

## Introduction

Bipolar disorder (BD) is associated with increased mortality^1^ and 8–12 years decreased life expectancy.^2^ Natural causes of death are the most prevalent causes of lost life years beginning early in life,^3^ likely because of increased comorbid medical burden from cardiovascular disease^4^ and diabetes^5^ that may partially be mediated through unhealthy lifestyle factors.^6^ Beyond these factors, mechanisms involved in accelerated aging in BD may contribute to the reduced lifespan^7^ observed. Although the pathophysiological background for BD and the associated reduced lifespan is largely unknown, increasing evidence derived from gene expression studies,^8^ investigation of post-mortem brain tissue^9^ and clinical studies^10^ indicates a role for oxidative stress as a relevant molecular mechanism.^11^ Damage to nucleic acids by oxidation, specifically, is linked with accelerated aging and has been demonstrated in several medical disorders associated with BD including diabetes,^12^ atherosclerosis^13^ and Alzheimer's disease.^14^ Further, oxidative damage to RNA was recently proposed as a novel disease mechanism contributing to medical diseases;^15^ however, *in vivo* studies in BD are limited, with only one study investigating urinary levels,^16^ which are considered more reliable than plasma levels.^15^

The prototypical oxidation lesions of DNA and RNA are constituted by 8-hydroxylation of guanine and measured reliably in the urine as the excretion products 8-oxo-deoxyguanosine (8-oxodG) and 8-oxo-guanosine (8-oxoGuo), respectively. Measureable by chromatographic methods, measurements of 8-oxodG and 8-oxoGuo in the urine provide a quantitative estimate of whole-body damage to DNA and RNA by oxidation *in vivo*.^17^

We recently demonstrated marked increases in both urinary 8-oxodG and 8-oxoGuo levels in euthymic patients compared with healthy control subjects, with increases of 40% and 43%, respectively.^16^ The increased levels of both markers were present through all affective phases, however, with no statistically significant difference between affective states. We speculated that the relatively low number of samples obtained in a manic or mixed state, which were additionally of relatively mild severity, could have contributed to the lack of statistical significance in comparisons between affective states, possibly constituting a type II error.

Consequently, in the present longitudinal study patients with BD type I in a current manic or mixed stated were followed for 6 months, and measurements of urinary levels of 8-oxodG and 8-oxoGuo were obtained in subsequent affective episodes or remission. We hypothesized that levels of urinary markers of DNA and RNA oxidation are increased in current affective states (hypomanic, manic, depressive and mixed states) compared with euthymia, and that markers of DNA and RNA oxidation are increased in patients with BD overall compared with levels in healthy control subjects. Lastly, we hypothesized that levels of DNA and RNA damage by oxidation decrease in patients remitting from an acute manic or mixed episode.

To the best of our knowledge, this is the first study investigating state-specific, intra-individual alterations in urinary markers of nucleoside damage generated by oxidation strictly in patients with BD I in a manic or mixed episode and in subsequent remission or depression.

## Materials and methods

We conducted a multicenter, longitudinal, clinical study investigating damage generated by oxidation of nucleosides in patients with BD I in an acute manic or mixed episode and during follow-up compared with repeated measures in healthy control individuals.

The study was approved by the Committee on Health Research Ethics of the Capital Region of Denmark (protocol no.: H-4-2012-114) and was conducted from December 2012 to December 2014 at Psychiatric Center Copenhagen, Copenhagen University Hospital, Denmark. Written informed consent was obtained from all participating subjects. The study complied with the Declaration of Helsinki.

### Participants

#### BD patients

Fifty-four patients with a potential diagnosis of BD І were recruited while being hospitalized with a manic or mixed episode at one of six psychiatric centers in the Mental Health Services—Capital Region of Denmark. Inclusion criteria were as follows: adults aged 18–60 years and ICD-10 (ref. 18) diagnosis of BD, type I. Exclusion criteria were as follows: severe medical disorder, substance abuse, pregnancy and insufficient Danish language skills. No subjects were recruited if they were subjected to restraint.

#### Healthy control subjects

Thirty-five healthy control subjects were recruited via the Blood Bank at Rigshospitalet, Copenhagen University Hospital, Denmark, by approaching blood donors in the waiting room on random occasions. The inclusion criteria were as follows: women and men, aged 18–60 years, no history of psychiatric illness and no first-generation family history of psychiatric illness. Exclusion criteria were identical to those applied to BD patients.

### Study design

Patients were followed prospectively during a 6–12-month period during and following discharge from the psychiatric ward. Contact with the patients was remained through regular phone calls (minimum once every fortnight), and they were instructed to contact the investigator on new signs of depressive or manic symptoms. The clinical state of the patients was monitored continuously through the patients' medical records. Clinical assessments and collection of urine samples were carried out when patients experienced a new affective episode and/or after return to euthymia. Affective episodes (hypomanic, manic, depressive and mixed) were defined according to the ICD-10 diagnostic criteria, and remission was defined according to the ICD-10 diagnostic criteria applying a revised duration criteria of 2 weeks.

The study was naturalistic, with patients receiving treatment as usual and with no influence from the study investigators.

Healthy control subjects were evaluated with clinical assessments and collection of urine samples on two separate occasions ~3 months apart.

In case of clinical signs of acute infection or other acute medical condition, assessment and biochemical analysis were postponed.

### Clinical assessments

All participants were assessed with standardized semi-structured interviews. The Schedules for Clinical Assessment in Neuropsychiatry interview^19^ was used to confirm the diagnosis of BD I using all available case materials, hospital records and interviews with the participant. Regarding the healthy control subjects, absence of lifetime psychiatric morbidity was confirmed at baseline and at follow-up according to the Schedules for Clinical Assessment in Neuropsychiatry interview.

At each assessment, the present clinical state (euthymia, hypomanic, manic, depressive and mixed episodes) of all participants was established according to ICD-10, concurrently with the collection of samples for laboratory analysis. The severity of depressive and manic symptoms was assessed using the 17-item Hamilton Depression Rating Scale^20^ and the Young Mania Rating Scale,^21^ respectively.

### Laboratory methods

When possible, the participants were fasting from midnight until blood and urine samples were collected the following day between 0800 and 1000 hours after a minimum of 15 min rest. The fasting state was recorded.

#### Blood collection

Standard biochemical parameters including hematological parameters, blood glucose, C-reactive protein, thyroid hormones, lipid status, ions, metabolites and liver enzymes were monitored to assure that these parameters were within the reference range during the study period.

#### Urine collection and preparation

A freshly voided spot urine sample was obtained using a standard sampling kit without any additives (In Vitro, Fredensborg, Denmark). The sample was kept on ice and centrifuged at 4 °C and 1590 *g* for 15 min, after which aliquots of 1.5 ml were transferred to Eppendorf tubes and stored at −80 °C until analysis.

#### Urinary 8-oxodG and 8-oxoGuo

The frozen samples were thawed, mixed and heated to 37 °C for 5 min and then centrifuged at 10 000 *g* for 5 min. The supernatant was used for the analysis. Samples from the same participant were grouped together on the same run in a randomly assigned sequence, with an even distribution of samples from patients and healthy control subjects across the assay. All samples were analyzed in two sessions on two adjacent days. The urinary content of the oxidized nucleosides 8-oxodG and 8-oxoGuo was quantified using a modified ultraperformance liquid chromatography and mass spectrometry assay previously used by our group,^16^ and described in detail elsewhere.^22^ The 8-oxodG and 8-oxoGuo urinary excretions were normalized to the urinary creatinine concentration, quantified by Jaffe's reaction. Laboratory personnel performing the analysis were blinded to the category of participants and the clinical state of patients with BD. The average within-day and between-day variations (relative s.d., %) estimated from the method validation were 3.8 and 7.4% for 8-oxodG and 2.3 and 9.0% for 8-oxoGuo, respectively.

### Statistical analyses

For the main analysis we employed a linear mixed effects model with random intercept for each participant, structured as a two-level model specifying a correlation of samples within participants. Level one represented within individual variation through repeated measures of 8-oxodG and 8-oxoGuo, and level two represented between individual variation. All analyses involved an unadjusted model and an adjusted model, with age and gender entered as covariates along with smoking, alcohol consumption and body mass index (BMI), and included a random intercept to accommodate correlations in the outcome variables over time within each participant. All other covariates were specified as fixed effects.

For all parametric tests, levels of 8-oxodG and 8-oxoGuo were transformed by the natural logarithm. Results of mixed model analyses are presented as backtransformed values represented by the parameter estimate *b,* expressing the ratio between groups. The level of statistical significance was set at *P<*0.05. The assumptions of linearity, independence of errors, homoscedasticity and normality were met. The statistical analysis was conducted with SPSS, version 20.0 (IBM, New York, NY, USA).

## Results

### Demographic and clinical characteristics

Demographic and clinical characteristics of the study participants are presented in Table 1.

Eleven of the BD patients received treatment for minor somatic disease (eight patients were treated for hypertension, two were treated for both hypertension and diabetes mellitus type II and one was treated for hypothyroidism).

The number of samples collected, symptom severity and urinary levels of 8-oxodG and 8-oxoGuo in relation to current affective state are presented in Table 2.

### Nucleoside damage from oxidation in patients with BD compared with healthy control subjects

Levels of 8-oxodG and 8-oxoGuo were increased 28% (*b*=1.28, 95% confidence interval (CI): 1.10–1.48, *P*=0.002) and 49% (*b*=1.49, 95% CI: 1.36–1.64, *P<*0.0001), respectively, in patients with BD overall compared with healthy control subjects. Considering only euthymic BD patients, levels of 8-oxodG were increased 22% (*b*=1.22, 95% CI: 1.03–1.44, *P*=0.021) and levels of 8-oxoGuo were increased 45% (*b*=1.45, 95% CI: 1.31–1.60, *P<*0.0001) compared with healthy control subjects.

**Figure 1:**
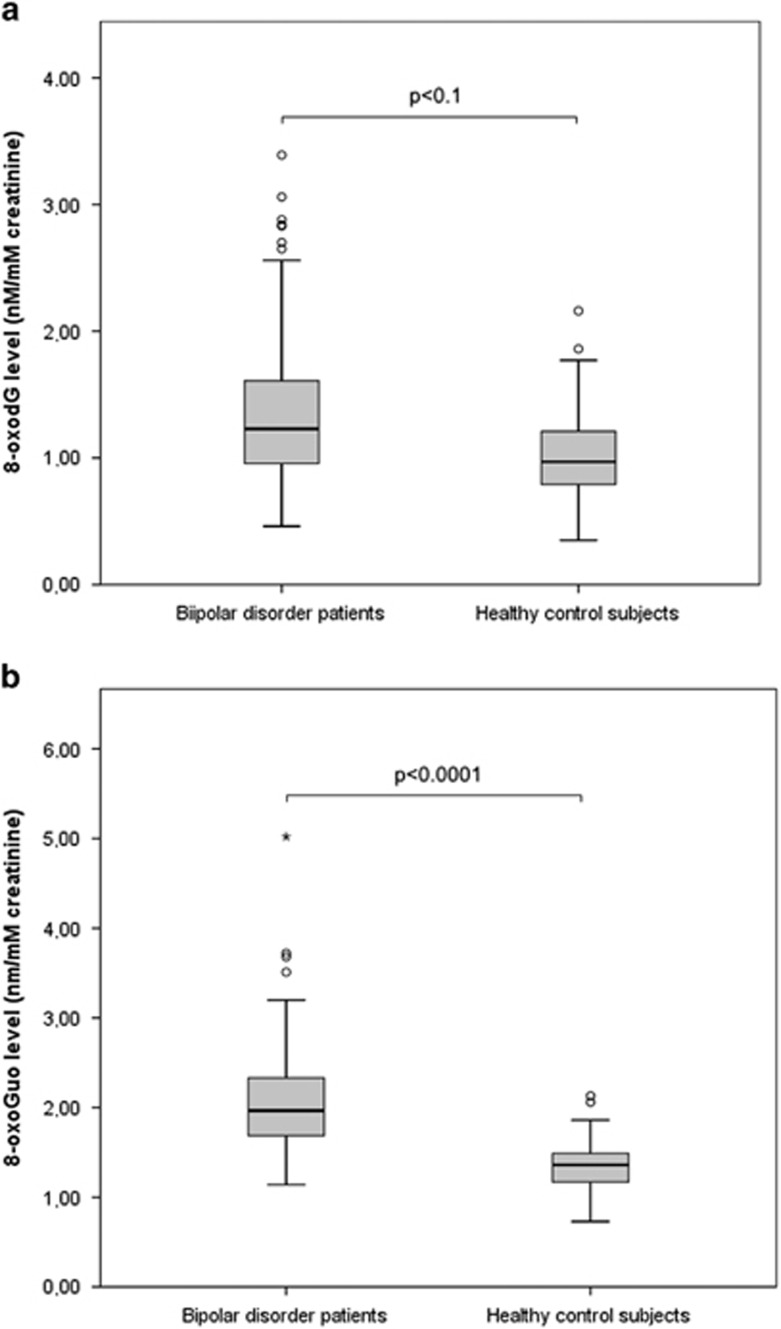
Box plot of urinary creatinine-corrected levels of 8-oxodG (**a**) and 8-oxoGuo (**b**) in patients with bipolar disorder overall (*n*=54) compared with healthy control subjects (*n*=35). The box shows the distribution of 50% of the sample around the median scores, with the extended lines showing the distribution of the lowest and highest 25% of scores, respectively. Circles and asterisks constitute outliers. Mixed model analysis adjusted for age, gender, smoking, alcohol intake and body mass index.

Adjusting for BMI, alcohol intake and smoking in addition to gender and age, the increase in levels of 8-oxoGuo in BD patients overall compared with healthy control subjects remained statistically significant with an increase of 34% (*b*=1.34, 95% CI: 1.19–1.51, *P<*0.0001), although this was not the case for 8-oxodG (*b*=1.16, 95% CI: 0.96–1.41, *P*=0.1). Similarly comparing only patients with BD І in an euthymic state with healthy control subjects, the increased levels remained statistically significant for 8-oxoGuo, with an increase of 36% (*b*=1.36, 95% CI: 1.20–1.54, *P<*0.0001), but not for 8-oxodG (*b*=1.13, 95% CI: 0.91–1.39, *P*=0.3), when adjusting for gender, age, BMI, alcohol intake and smoking (Figure 1).

In exploratory analyses excluding BD patients with minor somatic disease (*n*=11) or further adjusting for fasting state, results did not change (data not shown).

### State-specific alterations of nucleoside damage from oxidation in patients with BD І

**Figure 2:**
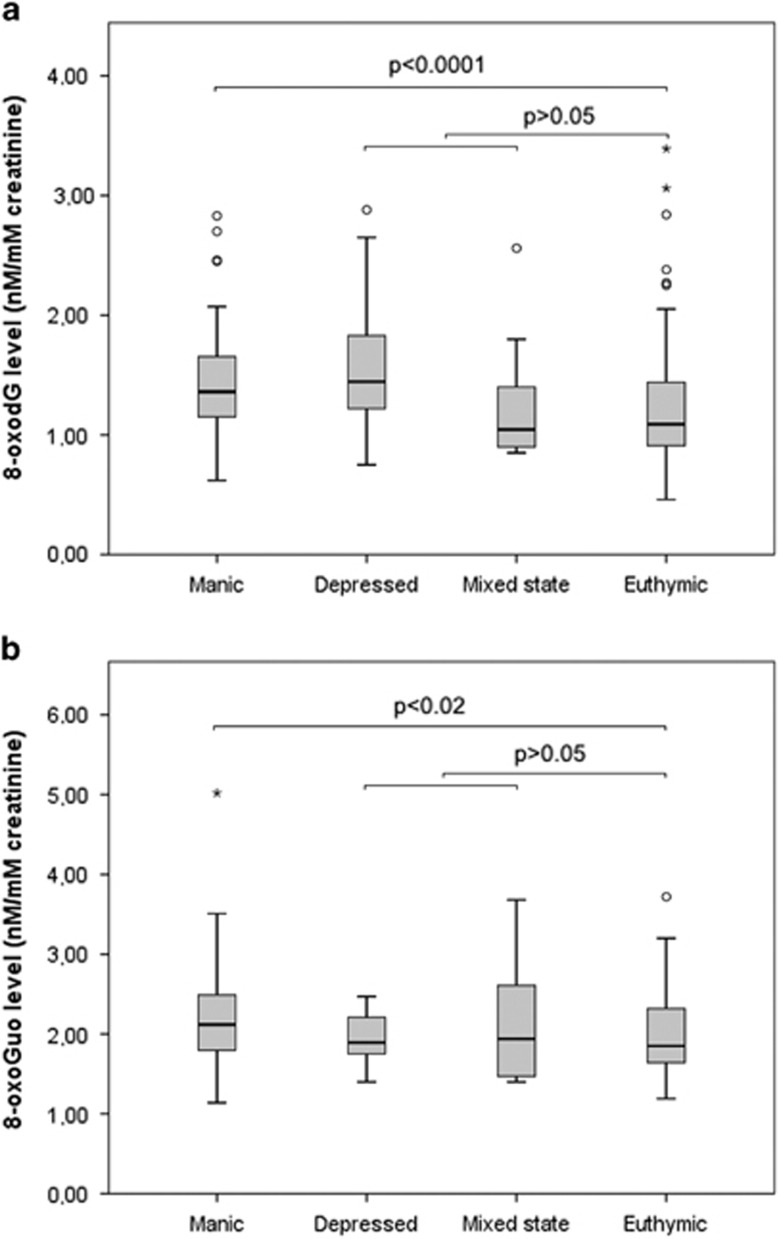
Box plot of urinary creatinine-corrected levels of 8-oxodG (**a**) and 8-oxoGuo (**b**) in patients with bipolar disorder according to the affective state (*n*=54). The box shows the distribution of 50% of the sample around the median scores, with the extended lines showing the distribution of the lowest and highest 25% of scores, respectively. Circles and asterisks constitute outliers. Mixed model analysis adjusted for age, gender, smoking, alcohol intake and body mass index.

8-oxodG and 8-oxoGuo levels in manic or hypomanic states were increased 17% (*b*=1.17, 95% CI: 1.09–1.25, *P<*0.0001) and 10% (*b*=1.10, 95% CI: 1.04–1.17, *P*=0.002), respectively, compared with an euthymic state. The differences remained statistically significant when adjusting for gender, age, BMI, alcohol intake and smoking, with an increase in 8-oxodG levels of 18% (*b*=1.18, 95% CI: 1.09–1.27, *P<*0.0001) and an increase in 8-oxoGuo levels of 8% (b=1.08, 95% CI: 1.01–1.15, *P*=0.023; Figure 2).

There were no significant differences in levels of either of the two markers between an euthymic state and a mixed state or between an euthymic state and a depressive state in neither unadjusted nor adjusted models (all *P>*0.05).

In exploratory analyses, entering the continuous variable illness duration and the categorical variable current use of medication (antidepressant, anticonvulsant, antipsychotic and lithium; yes, no) in addition to current affective state, age and gender the increased levels observed in a manic or hypomanic state compared with an euthymic state remained largely unchanged for both 8-oxodG (*b*=1.17, 95% CI: 1.09–1.25, *P<*0.0001) and 8-oxoGuo (*b*=1.09, 95% CI: 1.03–1.16, *P*=0.005). There was no effect of either illness duration or medication use on either marker.

In *post hoc* analysis excluding the 11 patients receiving treatment for minor physical illness (eight with hypertension, two with hypertension and diabetes mellitus type II and one with hypothyroidism) did not change the findings (data not presented).

### Change in nucleoside damage from oxidation in patients with BD І from mania to remission

The change in levels of 8-oxodG and 8-oxoGuo from a manic or mixed state to partial and full remission was evaluated in patients experiencing no affective episodes between hospital admission in a manic or mixed state and subsequent remission (*n*=26).

**Figure 3:**
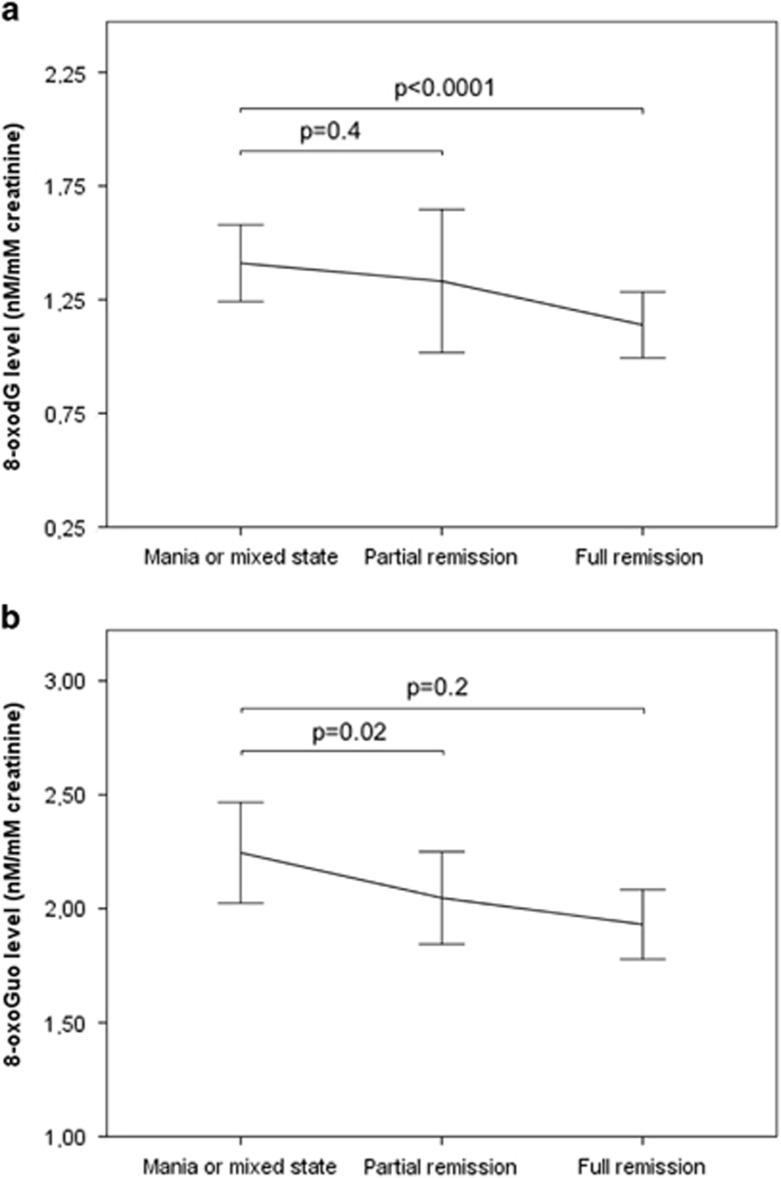
Urinary creatinine-corrected levels of 8-oxodG (**a**) and 8-oxoGuo (**b**) in patients with bipolar disorder hospitalized with a manic or mixed episode and subsequent partial and full remission (*n*=26). Data are expressed as mean±s.e.m. Mixed model analysis adjusted for age, gender, smoking, alcohol intake and body mass index.

Comparing levels of both markers in manic patients with levels in patients in subsequent remission in unadjusted analysis, levels of 8-oxodG decreased 14% (*b*=0.86, 95% CI: 0.79–0.93, *P<*0.0001) and levels of 8-oxoGuo decreased 10% (*b*=0.90, 95% CI: 0.83–0.96, *P*=0.003). The change in 8-oxodG levels remained statistically significant when adjusting for gender, age, BMI, alcohol intake and smoking, with a decrease of 15% (*b*=0.85, 95% CI : 0.77–0.94, *P<*0.0001) but not for 8-oxoGuo (*b*=0.95, 95% CI: 0.88–1.02, *P*=0.2; Figure 3).

A decrease in levels of both markers was observed from a manic or mixed state to partial remission; however, this was not statistically significant for either 8-oxodG (*b*=0.91, 95% CI: 0.77–1.07, *P*=0.2) or 8-oxoGuo (*b*=0.94, 95% CI: 0.81–1.08, *P*=0.4; Figure 3).

### Association between symptom severity and damage generated by oxidation of nucleosides

Adjusting for age and gender, there was no significant association between manic symptom severity according to the Young Mania Rating Scale scores and 8-oxodG (*b*=1.12, 95% CI: 1.00–1.02, *P*=0.08) or 8-oxoGuo (*b*=1.00, 95% CI: 0.99–1.01, *P*=0.8) in samples from patients with BD І in a manic or hypomanic state. Similarly, there was no significant association between depressive symptom severity measured by 17-item Hamilton Depression Rating Scale scores and 8-oxodG (*b*=1.0, 95% CI: 0.95–1.05, *P*=0.9) or 8-oxoGuo (*b*=1.01, 95% CI: 0.99–1.03, *P*=0.4) in samples from patients with BD І in a depressive state, adjusting for age and gender.

## Discussion

This study investigated, for the first time, state-specific, intra-individual alterations in urinary markers of nucleoside damage generated by oxidation strictly in patients with BD I included in a manic or mixed episode compared with repeated measurements in healthy control subjects. In accordance with our hypotheses, we found increased levels of RNA damage by oxidation by 34% in BD patients overall compared with healthy control subjects independently of minor physical illness and demographic and lifestyle factors such as smoking, alcohol consumption and BMI. Levels were increased by 36% in euthymic patients alone. Further, we found evidence of state-related alterations, with increased DNA and RNA damage by oxidation in patients with BD І in hypomanic and manic states compared with euthymia. Lastly, levels of both markers decreased in patients remitting from a manic or mixed episode, however, with changes remaining statistically significant after adjusting for age, gender, BMI, alcohol use and smoking for only 8-oxodG.

The present results confirm our previous findings of increased RNA damage by oxidation in BD patients.^16^ In contrast to our first study that included both BD I and II patients with relatively few numbers of hypomanic or manic episodes,^16^ we were here able to demonstrate state-related alterations, with increased levels of both the DNA and RNA markers in manic or mixed states compared with an euthymic state investigating a cohort of BD I patients. Urinary 8-oxodG and 8-oxoGuo appear more valid measures of total body damage to nucleosides by oxidation than their blood counterparts because these are mainly dependent on kidney function, whereas urinary levels, when corrected for creatinine concentration, are not.^23^ When comparing plasma levels of 8-oxodG and 8-oxoGuo between different individuals whose kidney function inherently differ, a potentially large bias could be introduced by measuring plasma values. Furthermore, there is no detailed empirical evidence for the use of plasma levels and the variability is unknown and may be large, thus limiting the validity,^23^ whereas the urinary DNA oxidation marker 8-oxodG has been extensively validated in multilaboratory exercises.^24^ Tissue measurement is also possible, however, reflecting the concentration of oxidized nucleosides in the biopsied tissue or harvested cells only. Beyond the present study and the previous study by our group,^16^ urinary levels of DNA and RNA damage have not been investigated in BD, and notably RNA damage by oxidation has not been investigated at all in either urine or blood. Two studies have investigated peripheral blood levels of nucleoside damage by oxidation. One found increased whole-blood 8-oxodG levels in patients with BD having a manic or depressed episode compared with healthy control subjects,^25^ and one found increased levels of a combined serum 8-oxodG and 8-oxoGuo markers in patients with BD in a manic or euthymic state compared with healthy control subjects.^26^ None of the studies adjusted the statistical analysis for potential confounders beyond age and gender, which was done in one study.^25, 27^

The finding of changes in DNA and RNA damage from an acute manic or mixed episode to remission suggests that oxidative damage to nucleosides may correspond with disease activity. Results suggested that these alterations may exhibit relatively large inter-individual variation; however, it is possible that individual trajectories are more uniform, and that oxidative DNA and RNA damages could have potential as more personalized markers of treatment response or symptomatic improvement.

In contrast to our previous finding in a cohort of patients with bipolar I and II rapid cycling disorder,^16^ our finding of increased nucleoside damage in patients with BD I compared with healthy control subjects only remained statistically significant for the RNA marker after adjusting for demographic and lifestyle variables. This could potentially be due to a type II error, given the lower overall number of urine samples in the present study despite the inclusion of a larger cohort. However, it also corresponds with findings in type 2 diabetes, where RNA damage, but not DNA damage was identified as an independent predictor of mortality and where changes in 8-oxoGuo during the first 6 years after diagnosis are also associated with mortality.^12^

The magnitude of RNA oxidation in BD patients was considerably larger than that recently demonstrated in patients with schizophrenia^28^ and depression.^29^ As previously speculated,^16^ this may be explained by the episodic nature of BD, in which the repeated need for adaption and re-setting of parameters^30^ due to recurring episodes may be especially detrimental to the maintenance of systemic homeostasis, possibly leading to higher levels of oxidative stress. RNA may be more vulnerable to oxidation than DNA, given its cytosolic location closer to mitochondria, the lack of protecting histones and its single-stranded nature.^15^ Oxidative modification of coding RNA leads to ribosomal dysfunction with the formation of non-functional or truncated proteins, and ultimately results in reduced numbers of functional proteins.^31^ Further, mistranslation of oxidized RNA may result in formation of mutated proteins, in turn potentially leading to incorrect folding of the protein,^32^ which may result in formation of aggregates that are the hallmarks of degenerative brain diseases, such as Alzheimer's disease.^33, 34^ Oxidative modification of RNA may thus constitute an epigenetic mechanism that could lead to severe and progressive disturbances of cellular function. We have therefore proposed RNA damage by oxidation as a contributing pathophysiological mechanism mediating the progressive nature of BD,^16^ with affective episodes and increasing illness duration leading to progressive shortening of inter-episode intervals and poorer treatment outcome.^35^ This mechanism could potentially further contribute to the increased risk of Alzheimer's disease observed in BD,^36^ which appears to increase with the number of affective episodes^37^ and to the increased medical burden overall observed in BD.^6^

Our findings strengthen the evidence of increased damage to nucleosides by oxidation in BD. Interestingly, examining multiple candidate genes in a cohort of patients with rapid cycling BD, we have recently found altered mRNA levels of three genes involved in mitochondrial function and DNA repair,^38^ further suggesting a role for disturbances related to these mechanisms in BD. Damage to nucleosides by oxidation is not specific for BD, having been identified in a number of both neuropsychiatric^28, 29, 32^ and medical disorders.^12, 39, 40^ This aspect is a limiting factor in the use of 8-oxodG and 8-oxoGuo as disease markers on their own; however, they may have potential use in addition to other proposed biomarkers.

Besides the advantage of using a longitudinal, repeated measures design for both BD patients and healthy control subjects, the present study benefited from a number of methodological strengths. These included standardization of urine sampling and laboratory analyses by obtaining samples in a 2-h interval in the morning, blinding of laboratory personnel to the clinical status of participants and by assigning samples randomly across assays in the analyses. We additionally adjusted statistical analyses for potential confounders including gender, age, smoking, alcohol consumption and BMI. These aspects of clinical studies of potential biomarkers in BD have been suggested to be considered for future studies by us^41^ and others.^42^ Whereas multiple comorbid medical conditions that are common in BD likely contribute to nucleoside damage by oxidation, the exclusion of patients with these conditions and the additional statistical control for potential confounders in the present study, diminish the likelihood of these factors influencing findings. There are limitations to the present study. First, patients with BD were all medicated and some patients suffered an unstable illness course during the study period and therefore changed medical treatment during the follow-up period. As lithium and mood stabilizers may have antioxidant properties, it therefore cannot entirely be excluded that medication at least partly explains the differences between healthy control subjects and also differences between affective states, although to a lesser degree. We did, however, not find any effect of medication in our analyses involving only BD patients, potentially indicating that medication did not influence results. Second, although our sample size was relatively large, the number of samples obtained may have been too low to detect a statistically significant difference in 8-oxodG levels between BD patients overall and healthy control subjects. Thus, the statistically nonsignificant results in this adjusted analysis and the analysis of change in 8-oxoGuo levels from mania to remission could potentially represent a type II error.

## Conclusion

In conclusion, this study demonstrated increased damage to RNA by oxidation in patients with BD І *in vivo* compared with healthy control subjects and state-related alterations with increased levels of damage to both DNA and RNA in manic and mixed states that decrease when achieving remission. The findings were independent of demographic and lifestyle variables and were not influenced by minor physical illness. The findings strengthen the evidence of oxidative stress in BD and support the role of RNA damage by oxidation in BD pathophysiology previously proposed by our group. The potential for urinary excretion of 8-oxodG and 8-oxoGuo to function as state and trait biomarkers as well as markers of risk and stage in BD should be further explored.

## Figures and Tables

**Table 1:** Demographic and clinical characteristics of study participants at inclusion

	*Bipolar disorder patients*	*Healthy control subjects*	*Statistics*	P-*value*
*N*	54	35		
Age (years)	41.911.9 (20–60)	36.7±11.6 (18–59)	*t*=2.06	0.042
Gender (male–female)	32/22	20/15	*X*^2^=0.039	0.843
Education (years total)	14.0±3.3 (8–22)	15.1±2.2 (10–20)	*t*=−1.763	0.08
Number of smokers (%)	31 (57.4)	7 (20)	*X*^2^=12.145	<0.0001
Alcohol intake >14 units per week (%)	4 (7.4)	3 (8.6)	*X*^2^=0.040	0.842
Body mass index	26.3±4.9 (20–43)	22.6 ±4.0 (17–35)	*t*=3.642	<0.0001
Blood glucose (mmol l^−1^)	5.3±0.8 (4.0–7.4)	5.0±0.8 (3.7–8.0)	*t*=1.526	0.131
Cholesterol (mmol l^−1^)	4.2±0.8 (2.7–6.0)	4.7±0.9 (3.1–6.7)	*t*=−2.829	0.006
HDL cholesterol (mmol l^−1^)	1.4-0.4 (0.6–2.3)	1.6±0.5 (0.9–2.8)	*t*=−2.157	0.034
LDL cholesterol (mmol l^−1^)	2.3±0.6 (1.2–3.6)	2.8±0.8 (1.3–4.7)	*t*=-3.167	0.002
Triglyceride (mmol l^−1^)	1.1±0.6 (0.5–2.9)	0.8±0.5 (0.3–2.4)	*t*=2.471	0.016
YMRS	23.8±6.4	1.0±1.7	*t*=20.471	<0.0001
HAMD-17	5.1±5.5	1.7±1.9	*t*=3.405	<0.0001
Duration of illness (years)	15±11.0 (0.06–42)			
Number of depressive episodes	3.7±4.0 (0–18)			
Number of hypomanic episodes	2.5±5.6 (0–25)			
Number of manic episodes	3.9±4.2 (0–23)			
Number of mixed episodes	0.4±0.9 (0–5)			
Number of hospitalizations	7.2±10.1 (1–67)			
Lithium treatment	27 (50.0)			
Anticonvulsant treatment	16 (29.6)			
Antipsychotic treatment	50 (92.6)			
SSRI treatment	1 (1.9)			
Newer antidepressant treatment	0 (0.0)			
Older antidepressant treatment	1 (1.9)			

Abbreviations: HAMD-17, 17-item Hamilton Depression Rating Scale; HDL, high-density lipoprotein; LDL, low-density lipoprotein; SSRI, selective serotonin reuptake inhibitor; YMRS, Young Mania Rating Scale.

Data are expressed as mean±s.d. (range) or number (percentage).

**Table 2:** Symptom severity and 8-oxodG and 8-oxoGuo levels

	*Healthy control subjects (*n=*70)*	*Bipolar disorder patients*			
		*Euthymic (*n=*77)*	*Depressive state (*n=*18)*	*Manic state (*n=*43)*	*Mixed state (*n=*10)*
HAMD-17	1.5±1.9 (0–9)	3.4±3.9 (0–14)	15.2±3.2 (10–22)	3.0±3.0 (0–13)	14.6±3.7 (9–22)
YMRS	0.8±1.4 (0–6)	1.5±2.5 (0–13)	1.2±1.8 (0–6)	23.6±7.3 (8–39)	17.7±4.5 (11–25)
8-oxodG	1.01±0.36	1.25±0.57	1.54±0.58	1.45±0.5	1.28±0.54
8-oxoGuo	1.36±0.28	1.98±0.5	1.92±0.3	2.2±0.7	2.12±0.75

Abbreviations: 8-oxodG, 8-oxo-deoxyguanosine; 8-oxoGuo, 8-oxo-guanosine; HAMD-17, 17-item Hamilton Depression Rating Scale; YMRS, Young Mania Rating Scale.

*N* represents number of samples. 8-oxodG and 8-oxoGuo levels are expressed as nm mm^−1^ creatinine.
